# Baseline [^68^Ga]Ga-PSMA-11 PET/CT before [^177^Lu]Lu-PSMA-617 Radioligand Therapy: Value of PSMA-Uptake Thresholds in Predicting Targetable Lesions

**DOI:** 10.3390/cancers15020473

**Published:** 2023-01-12

**Authors:** Daniel Groener, Sina Schneider, Justus Baumgarten, Christian Happel, Konrad Klimek, Nicolai Mader, Christina Nguyen Ngoc, Jennifer Wichert, Philipp Mandel, Nikolaos Tselis, Frank Grünwald, Amir Sabet

**Affiliations:** 1 Department of Nuclear Medicine, University Hospital Frankfurt, Theodor Stern Kai 7, 60590 Frankfurt, Germany; 2 Department of Urology, University Hospital Frankfurt, Theodor Stern Kai 7, 60590 Frankfurt, Germany; 3 Department of Radiation Oncology, University Hospital Frankfurt, Theodor Stern Kai 7, 60590 Frankfurt, Germany

**Keywords:** PSMA, [^177^Lu]Lu-PSMA-617, [^68^Ga]Ga-PSMA-11 PET/CT, metastatic castration-resistant prostate cancer

## Abstract

**Simple Summary:**

The prostate-specific membrane antigen (PSMA), a transmembrane protein frequently present on prostate cancer cells, has gained considerable interest as a target for both molecular imaging and therapy. Patients with metastatic castration-resistant prostate cancer can be successfully treated by delivery of beta particle-emitting ^177^Lutetium to the prostate-specific antigen in a therapeutic concept termed radioligand therapy. Imaging with positron emission tomography (PET) plays a crucial role in patient selection prior to radioligand therapy and subsequent molecular response assessment. The presented study aims to investigate the role of quantitative uptake parameters on baseline ^68^Gallium-PSMA-11 PET/CT imaging as to their association with lesion response to radioligand therapy at individual tumor sites. Special emphasis is placed on the utility of PSMA-uptake thresholds for pretherapeutic prediction of lesion response.

**Abstract:**

Baseline uptake on prostate-specific membrane antigen (PSMA)-targeted imaging is a prerequisite for radioligand therapy (RLT) with [^177^Lu]Lu-PSMA-617. This study aims to quantify lesion-based response to RLT in relation to pretreatment standard molecular imaging metrics derived from [^68^Ga]Ga-PSMA-11 PET/CT. Sixty-one patients with mCRPC underwent [^68^Ga]Ga-PSMA-11 PET/CT imaging before and after a median of 4 (IQR 2–6) RLT cycles. Maximum and mean standardized uptake values (SUV_max_, SUV_mean_), as well as tumor-to-liver ratio (TLR), were assessed. A median of 12 (IQR 7–17) lesions was analyzed per patient, resulting in a total of 718 lesions. Lesions with ≥30% SUV_max_ decline or falling below the blood pool uptake were considered responsive; ≥30% SUV_max_ increase marked lesion progression. Additionally, 4-point visual scoring was performed according to E-PSMA consensus. In total, 550/718 (76.6%) lesions responded to RLT, including 389/507 (76.7%) bone metastases and 143/181 (79.0%) lymph node metastases. Baseline SUV_max_, SUV_mean_, and TLR values were associated with lesion response by a moderate but significant correlation (r_s_ = 0.33, *p* < 0.001, r_s_ = 0.32, *p* < 0.001, and r_s_ = 0.31, *p* < 0.001, respectively). For the classification of lesion progression based on baseline PSMA uptake, receiver operating characteristics (ROC) found SUV_max_, SUV_mean_, and TLR to have comparable discriminatory value (AUC 0.85, 0.87, and 0.83). Of 42 tumor sites with baseline uptake below the liver (V-score < 2), 19/42 (45.2%) were responsive, 9/42 (21.4%) were stable, and 14/42 (33.3%) showed progression, leaving liver uptake a threshold with low prognostic value for the identification of RLT-refractory lesions (PPV 33%). This was observed accordingly for various liver uptake-based thresholds, including TLR < 1.5, <2.0 with a PPV at 24%, 20%, respectively. Standard uptake parameters quantified by routine baseline [^68^Ga]Ga-PSMA-11 PET/CT are moderately associated with post-treatment lesion response to [^177^Lu]Lu-PSMA-617. Commonly applied liver-based uptake thresholds have limited value in predicting refractory lesions at individual tumor sites.

## 1. Introduction

Radioligand therapy (RLT) has been increasingly adopted as a treatment option for men with metastatic castration-resistant prostate cancer (mCRPC). Recently, VISION (NCT03511664), an open-label, multicenter, phase 3 trial, yielded excellent anti-tumoral activity and tolerability of RLT with [^177^Lu]Lu-PSMA-617 in this subset of patients [1]. The concept of RLT in prostate cancer is marked by selective delivery of radionuclides to the type II transmembrane protein prostate-specific membrane antigen (PSMA). Overexpressed on the surface of prostate cancer cells, PSMA provides an ideal binding site for both selective radiotherapy and high-sensitivity molecular imaging, warranting a closely interrelated diagnostic and therapeutic process [2]. Normal organs, including salivary glands, kidneys, and liver, are known to exhibit PSMA expression to physiological extents and have recently gained interest as landmarks for semiquantitative stratification of lesion uptake [3].

PSMA-targeted positron emission tomography (PET) plays a pivotal role in the selection of patients best suited for RLT, as uptake kinetics on [^68^Ga]Ga-PSMA-HBED-CC ([^68^Ga]Ga-PSMA-11) PET/CT imaging may anticipate radioligand binding during RLT and local treatment efficacy [4,5]. Based on this reasoning, the EANM procedure guidelines for [^177^Lu]Lu-PSMA-617 has set out a lesion uptake exceeding the mean uptake of the liver to be considered a prerequisite for RLT [6,7]. Prospective trials have also adopted liver uptake-based inclusion criteria including VISION, where PSMA negativity was defined as a lesion uptake visually falling below the uptake of healthy liver tissue [1,8].

Various target lesion-based approaches have been proposed to assess response to RLT. It is understood that structural response assessment has limited value in mCRPC, with bone lesions being poorly measurable. Also, lymph node involvement and response are potentially underestimated by structural imaging alone [9]. The applicability of molecular response assessment to PSMA-targeted imaging is subject to investigation and has recently been supported by joint consensus [10,11,12]. Hallmarks of molecular response include a decline in standardized uptake and visual lesion uptake falling below organ-derived thresholds [11].

The objective of this study is to investigate the role of pretreatment PET/CT semi-quantitative uptake per lesion as a possible denominator of response to RLT at individual tumor sites. The location, visual uptake (V-score), standardized uptake values (SUV_max_, SUV_mean_), and tumor-to-liver ratio (TLR) of lesions are specifically taken into consideration. To meet this aim, a distinct set of target lesions of RLT patients is extracted on baseline PSMA-PET/CT imaging and subsequently analyzed intra-individually and per lesion.

## 2. Materials and Methods

### 2.1. Patients

A total of 61 patients with mCPRC was included in this retrospective single-center series. Inclusion criteria mandated that patients receive [^68^Ga]Ga-PSMA-11 PET/CT following the same scanning protocol at baseline and after RLT with [^177^Lu]Lu-PSMA-617. Included patients were required to have a minimum of three assessable lesions, and no new or change in disease-modifying medication (first- or second-generation antiandrogenes, taxane-based chemotherapy) between the two performed scans. Indications for RLT were confirmed by a multidisciplinary team, including board-certified nuclear medicine physicians, urologists, radiation oncologists, pathologists, and oncologists. Production and administration of [^68^Ga]Ga-PSMA-11 PET/CT and [^177^Lu]Lu-PSMA-617 were performed in accordance with legal regulations set out in the German Drug Registration and Administration Act (AMG § 13 2b).

### 2.2. Radiopharmaceutical Synthesis and PET/CT Imaging Procedure

Radiolabeling of PSMA-11 followed an established protocol [13]. ^68^Gallium was obtained from a ^68^Ge/^68^Ga radionuclide generator (GalliaPharm, Eckert & Ziegler Radiopharma, Berlin, Germany). [^68^Ga]Ga-PSMA-11 was administered by intravenous injection and target activity per patient was 1.8–2.5 MBq/kg body weight. Whole-body images (vertex to mid-thigh) were acquired 61 ± 12 min after tracer injection and PET acquisition time was 4 min per bed position. CT data were acquired for attenuation correction and anatomical localization using an X-ray tube voltage of 130 kV with a modulated tube current (CARE Dose 4D, Siemens, Erlangen, Germany). Acquisitions were carried out on a Biograph 6 PET/CT scanner (Siemens, Erlangen, Germany), with decay, scatter, and attenuation correction performed in accordance with the procedure guidelines set out by the joint EANM and SNMMI consensus statement [14].

### 2.3. PET/CT Imaging Assessment

Image reconstruction was carried out using dedicated manufacturer workstations and software (Syngo TrueD, Vers 61.b, Siemens Healthcare, Erlangen, Germany). PET data was reconstructed applying an iterative ordered subset expectation maximization (OSEM) algorithm with four iterations and eight subsets; gaussian filtering was used. The matrix size was 168 × 168 at a 5 mm slice thickness. For further analysis, PET and CT dataset fusion was performed using a commercial software package (OsiriX MD Version 10.0.4, Pixmeo, Bernex, Switzerland). In each patient a set of lesions was segmented, based on the following inclusion criteria: non-physiological, site-specific uptake greater than in surrounding background tissue, and detectability on co-registered computed tomography. At each site of disease involvement, i.e. skeletal, lymph nodal, visceral, and primary/locally recurrent disease sites, up to 10 lesions were chosen, including the lesion with the highest and lowest uptake. Maximum standardized uptake value (SUV_max_) and mean standardized uptake value (SUV_mean_) within a 40% isocontour were registered for all lesions. Mean standardized liver uptake (SUV_mean_ liver) was determined by placing a 30 mm spherical volume of interest (VOI) into a non-affected portion of the right liver lobe. For further analysis, the tumor-to-liver uptake ratio (TLR) was calculated for each lesion by dividing SUV_max_ by the mean liver uptake within the above VOI. All lesions were also classified on a visual 4-point scale according to recently met E-PSMA consensus [11]: (0) below blood pool, (1) > blood pool ≤ liver uptake, (2) > liver uptake ≤ parotid uptake, (3) > parotid uptake. Lesions were defined as responsive lesions (RL) if showing a ≥30% decline in SUV_max_ or when falling below a visually detectable threshold (V-score 0 based on E-PSMA criteria) on posttherapeutic PSMA PET/CT assessment. The remaining lesions were classified as stable lesions (SL) with an SUV_max_ change <30% and progressive lesions (PL) with an SUV_max_ increase ≥30% [15,16].

### 2.4. ^177^Lu-PSMA-617 Radiolabeling and Administration

Radiolabeling of PSMA-617 with ^177^LuCl_3_ was carried out as previously described in detail [17,18]. [^177^Lu]Lu-PSMA-617 was administered by slow intravenous injection over 30–60 s, preceded and followed by 1000 mL of saline infusion. RLT was performed as an inpatient procedure at the nuclear medicine therapy ward in accordance with radioprotection regulations. Six cycles with an activity of 7.4 GBq per cycle were intended; administered activities were modified in patients with potential risks for toxicity.

### 2.5. Statistical Analysis

Results are presented as median with interquartile range (IQR) and mean ± standard deviation (SD) for continuous variables. Categorical variables are reported as frequencies with respective percentages. Comparison of means was performed by a paired t-test for intraindividual analysis or by using a Mann-Whitney U test if data were not normally distributed. Association of categorical parameters was analyzed using non-parametric rank correlation (Spearman’s correlation coefficient denoted with r_s_). Receiver operating characteristics (ROC) analysis was applied to determine the ability of baseline PET/CT metrics (SUV_max_, SUV_mean_, TLR) to predict treatment lesion progression at individual sites. The area under the curve (AUC) was calculated for all lesions and separately for bone metastases and lymph node metastases. Site-specific cutoff values for the detection of PL were calculated using Youden’s J statistic. Various liver-based thresholds were tested as to their discriminatory value for lesion progression. Odds ratios (OR) were calculated with 95% confidence intervals (CI). Statistical analyses were performed with SPSS (version 27.0, IBM, Armonk, NY, USA) and GraphPad Prism (version 9.1.1, GraphPad Software, San Diego, CA, USA). All tests were two-sided, with *p*-values < 0.05 denominating statistical significance.

## 3. Results

Overall, 718 lesions were included in the analysis, consisting of 507 bone, 181 lymph node, 22 visceral, and 8 primary/locally recurrent sites in 61 patients with mCRPC (median age 72 [IQR 67–78] years). This corresponded to a median 12 (IQR 7–17) lesions per patient. Patient characteristics at baseline are detailed in Table 1. All patients received baseline [^68^Ga]Ga-PSMA-11 PET/CT imaging and subsequently underwent a median of 4 (IQR 3–6) cycles of [^177^Lu]Lu-PSMA-617 given with a mean treatment activity of 6.9 ± 1.4 GBq per cycle. Cumulative activity per patient was 29.0 ± 17.5 GBq. Of all patients, 31/61 (50.8%) showed ≥50% PSA decline 12 weeks after treatment initiation, while 15/61 (24.6%) showed PSA progression based on PCWG3 criteria (≥25% PSA increase).

**Figure 1:**
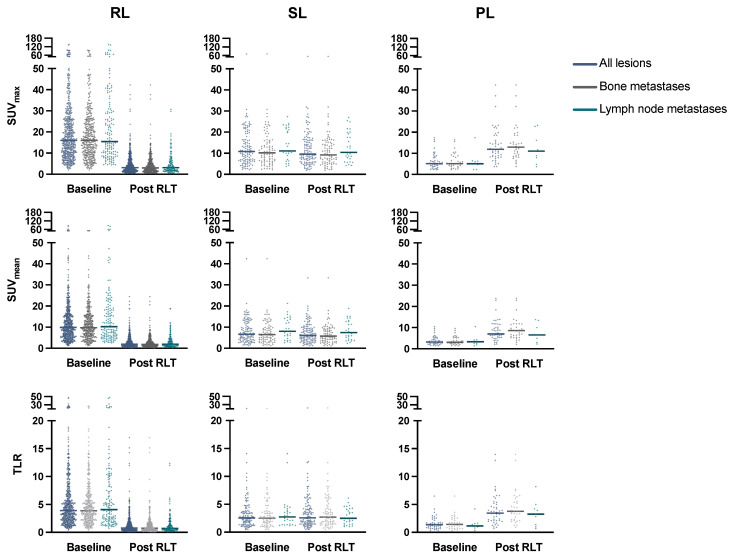
Change in SUV_max_, SUV_mean_, and TLR in all lesions, and in bone/lymph node metastases subgroups with median values (line). RL: responding lesions, SL: stable lesions, PL: progressive lesions, RLT: radioligand therapy, SUV: standardized uptake value, TLR: tumor-to-liver ratio.

**Figure 2:**
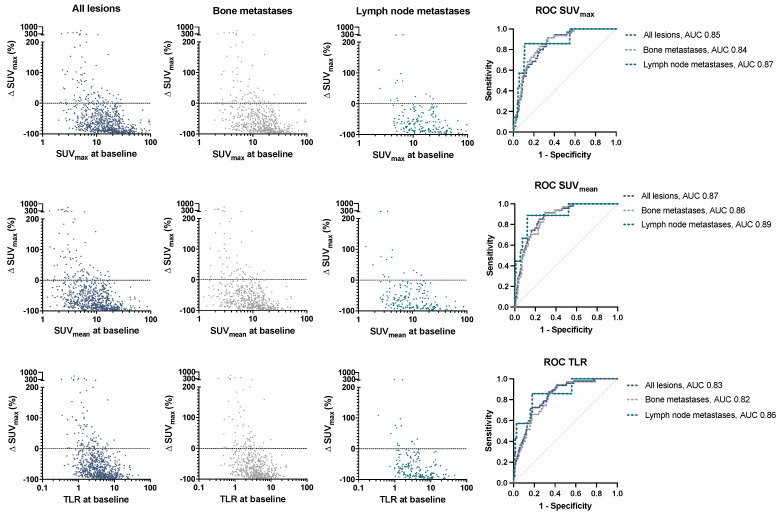
Change (%) in SUV_max_ over baseline values for SUV_max_, SUV_mean_, and TLR in all lesions and in bone/lymph node metastases. Receiver operating characteristic (ROC) curve for lesion progression depending on baseline PET values. SUV: standardized uptake value, TLR: tumor-to-liver ratio, AUC: area under the curve.

Details on lesion characteristics are provided in Table 2. At baseline, all assessed lesions showed a median SUV_max_ of 14.11 (IQR 8.25–23.01) and SUV_mean_ of 8.72 (IQR 5.09–14.39); tumor-to-liver ratio (TLR) was 3.36 (IQR 1.98–5.70). Of all lesions, 550/718 (76.6%) responded to RLT, consisting of 389/507 (76.7%) bone metastases and 143/181 (79.0%) lymph node metastases. There was no significant difference in mean SUV_max_ decline in bone vs. lymph node metastases (*p* = 0.23). Responding lesions (RL) had significantly higher SUV_max_, SUV_mean_, and TLR values at baseline than stable (SL) or progressive lesions (PL), with an SUV_max_ at 16.01 (RL), 10.82 (SL), 5.11 (PL) (*p* < 0.001), SUV_mean_ at 9.88 (RL), 6.62 (SL), 3.18 (PL) (*p* < 0.001), and TLR at 3.89 (RL), 2.55 (SL), 1.36 (PL) (*p* < 0.001). This was observed in both lymph node and bone metastases (Figure 1, Table 2). Visceral and locally recurrent/primary tumor sites were spared from subgroup analysis due to limited sample size. Baseline values for SUV_max_, SUV_mean_, and TLR were associated with lesion response after RLT by a moderate but significant correlation (r_s_ = 0.33, *p* < 0.001, r_s_ = 0.32, *p* < 0.001, and r_s_ = 0.31, *p* < 0.001, respectively); the relationship is shown in Figure 2.

**Figure 3:**
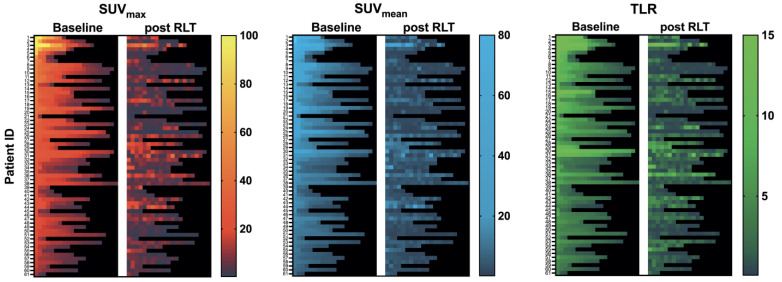
Heatmap of all assessed lesions: SUV_max_, SUV_mean_, and TLR at baseline and after RLT per patient. Rows correspond to treated patients sorted by intensity (baseline SUV_max_) of the hottest lesion assessed; columns represent lesions sorted from hottest lesion (baseline SUV_max_) per patient to coldest lesion (left to right).

**Figure 4:**
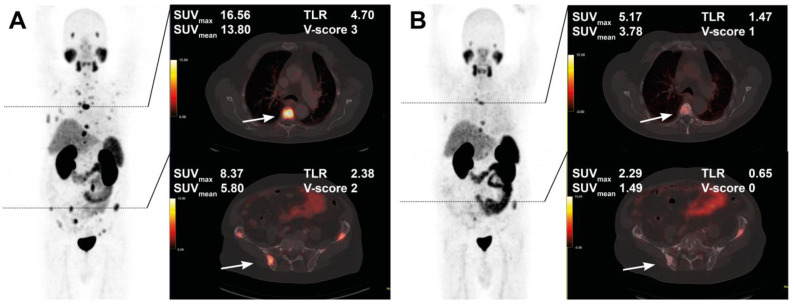
Treatment response in a 76-year-old patient after 4 cycles of RLT with cumulative 32.0 GBq [^177^Lu]Lu-PSMA-617. Maximum intensity projection (MIP) images (left) with cross-section fusion images in a thoracic and pelvic plane at baseline (**A**) and after RLT (**B**). Lesions (white arrows) show response to RLT, with the upper lesion in the thoracic spine maintaining detectable residual activity.

**Figure 5:**
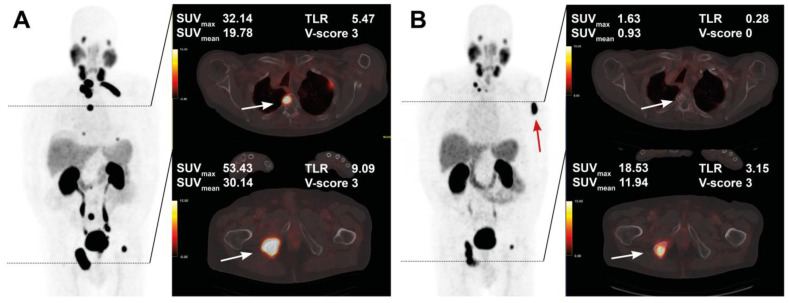
Pre- and post-treatment images in an 83-year-old patient after 5 cycles of RLT with cumulative 35.1 GBq [^177^Lu]Lu-PSMA-617. Maximum intensity projection (MIP) images (left) with cross-section fusion images in a thoracic and pelvic plane at baseline (**A**) and after RLT (**B**). Lesions (white arrows) with intense baseline uptake showing response to RLT; a new bone metastasis develops in the left humerus (red arrow).

The course of all lesions per patient is depicted in Figure 3. In 11 patients showing lesion response in included baseline tumor sites, ≥2 new onset lesions on posttherapeutic imaging led to discontinuation of RLT. Examples of intraindividual lesion response on [^68^Ga]Ga-PSMA-11 PET/CT imaging are provided in Figure 4 and Figure 5.

### 3.1. ROC Analysis

ROC analysis was performed to classify lesion progression based on pretherapeutic SUV_max_, SUV_mean_, and TLR values. The area under the curve (AUC) was comparable for SUV_max_, SUV_mean_, and TLR with 0.85, 0.87, and 0.83, respectively (Figure 2). Cutoff values were calculated to separate PL at individual tumor sites based on PET parameters: a baseline SUV_max_ value of 6.49 was found to discriminate PL from SL and RL (87% sensitivity, and 74% specificity), with the corresponding SUV_mean_ and TLR thresholds at 4.85 (80% sensitivity, 77% specificity) and 1.76 (82% sensitivity, 77% specificity). While liver-based thresholds allowed the stratification of lesions based on their response category in a balanced manner, their value is limited for prognostication, as shown for various liver-derived thresholds in Table 3.

### 3.2. V-Score of Lesions

**Figure 6:**
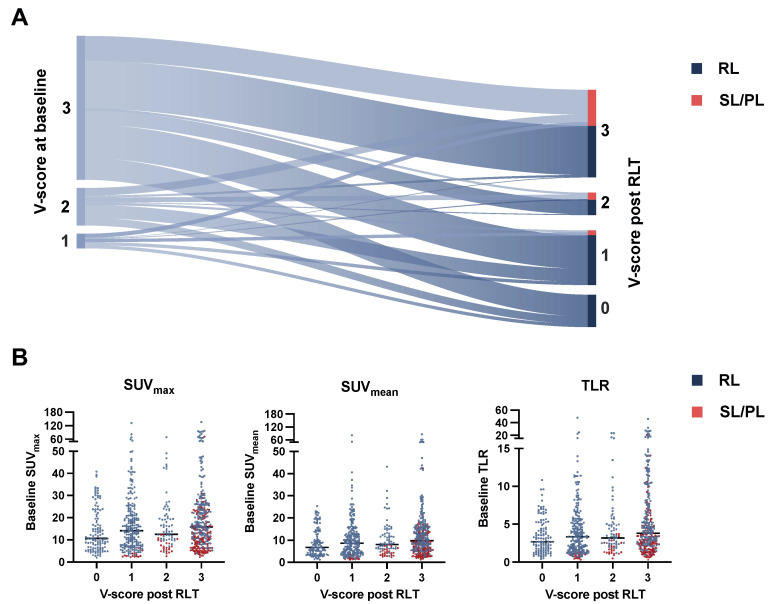
Sankey diagram with change in V-score from baseline to posttherapeutic imaging (**A**). Beeswarm plot stratifying baseline PET metrics (SUV_max_, SUV_mean_, TLR) with median values (line) by posttherapeutic V-score and lesion response (**B**). RL: responding lesions, SL: stable lesions, PL progressive lesions, SUV: standardized uptake value, TLR: tumor-to-liver ratio.

In addition to semiquantitative PET measurements, visual scoring (V-score) was noted for all lesions. The V-score change from baseline to posttherapeutic PET assessment was registered, as depicted in Figure 6A. At baseline, 676/718 (94.2%) lesions had an uptake higher than the liver threshold (V-scores ≥ 2); 42/718 (5.8%) had a V-score < 2. On PET imaging after RLT, of all responding lesions 118/505 (21.5%) declined to non-detectable uptake values (V-score 0), 230/505 (41.8%) while responsive lesions showed V-scores ≥ 2, thus exceeding the liver threshold. Of the 42 lesions included with a baseline V-score < 2, 19/42 (45.2%) showed response to RLT marked by either significant decline in uptake (SUV_max_ decline ≥ 30%) or non-detectability; 28/42 (66.7%) lesions were non-progressive (i.e. SL/RL). Lesions declining to an uptake below the blood pool after RLT (V-score = 0) had lower uptake values at baseline than lesions showing posttherapeutic V-scores ≥ 1 with SUV_max_ 10.63 vs. 14.89 (*p* = 0.002), SUV_mean_ 6.78 vs. 9.15 (*p* = 0.009), and TLR 2.71 vs. 3.48 (*p* < 0.001) (Figure 6B). A minor numer of lesions (six in total) showed response to treatment even though an increase in V-score was noted, attributable to an intraindividual shift in organ uptake. On matched comparison of the entire cohort, mean liver uptake was not significantly changed after RLT compared to baseline values (4.07 vs. 4.14, *p* = 0.09).

## 4. Discussion

This study indicates that routine PET parameters assessed by [^68^Ga]Ga-PSMA-11 PET/CT at baseline are moderately associated with lesion response to [^177^Lu]Lu-PSMA-617 RLT. Low uptake lesions were more frequently subject to progression, whilst addressable by RLT in a significant fraction of cases. Baseline metrics and liver-based thresholds had only limited value for single-lesion response prediction.

The concept of RLT is marked by the targeted delivery of alpha or beta particle-emitting radionuclides to the surface of prostate cancer cells. Preclinical findings in mice receiving [^177^Lu]Lu-PSMA-617 RLT have proven that a higher density of the transmembrane glycoprotein PSMA on prostate cancer cells is associated with increased ligand internalization and treatment efficacy [19]. Cytotoxic effects are exerted by beta-emitter radiation which induces dose-dependent single- and double-strand DNA breaks once sufficient intracellular accumulation is reached [20].

In patients treated with RLT, dosimetry studies have shown an association of pretreatment imaging parameters and locally absorbed doses during subsequent treatment cycles. Peters et al. reported absorbed dose estimates derived from pretherapeutic PSMA PET/CT in tumor sites and normal organs (n = 22) of RLT patients to be correlated with intratherapeutic absorbed doses [5]. However, so far, subsequent response to RLT has rarely been addressed from a single lesion-based perspective. Stangl-Kremser et al. found a significant association of immunohistochemically proven PSMA expression with SUV_max_ values on PET/CT imaging in biopsied prostate cancer metastases. A single-lesion analysis of response to RLT was then conducted in 9 lesions (5 responding lesions with SUV_max_ decline ranging from −33 to −43%), where no association could be established to baseline features, most likely due to the limited sample size [15]. Recently, van der Sar et al. analyzed 237 lesions in 32 patients in both lesion-based and patient-based approaches, finding higher SUV_peak_ values of the most avid metastasis per patient to be predictive of imaging-based response (total lesion-PSMA decline ≥30%) to 2 cycles of RLT [21]. With imaging-based partial response per lesion (iPR) defined as ≥30% SUV_peak_ decline and complete lesion response as a decline below the blood pool (iCR), 149/237 (63%) lesions were classified as responsive, compared to 550/718 (77%) in our cohort. Tumor sites with iPR had higher SUV_max_ baseline values compared to stable (iSD) and progressive lesions (iPD), with geometric means at 17.96, 8.58, and 4.99. These results are in line with our findings from a considerably larger cohort, with median SUV_max_ values for RL, SL, and PL at 16.01, 10.82, and 5.11, respectively. In both our study and their cohort, lymph node metastases showed a tendency towards higher baseline SUV_max_ values compared to bone metastases, while response rates did not differ significantly. In our cohort, lymph node and bone lesions showed comparable rates of response (79 vs. 77%) and no difference in SUV_max_ decline percentage (*p* = 0.23). Previously reported superior RLT response in patients with lymph node-dominant tumor burden in comparison to patients with mixed patterns of metastatic disease could therefore be attributable to differences in disease phenotype or tumor burden rather than to inferior activity of RLT in bone metastases [22,23].

PSMA PET/CT is the gold standard for screening patients prior to RLT and has been named a prerequisite for inclusion into several prospective trials, including VISION (NCT03511664) [1,7,24]. Here, a tumor lesion uptake exceeding the liver uptake is considered an inclusion criterion for patients. In their landmark prospective study, Hofman et al. selected patients for tumor sites showing an SUV_max_ at least 1.5 times the normal liver uptake, whilst excluding FDG-positive disease with low PSMA expression by dual FDG and PSMA-PET/CT baseline imaging [7]. Sartor et al. (VISION) mandated a visual uptake superior to the liver uptake in all lesions which were also subject to RECIST evaluation based on size criteria [1,8]. Liver-derived thresholds are also applied to select patients for peptide receptor radionuclide therapy (PRRT) in neuroendocrine tumors (NET) [25]. In a retrospective series, Kratochwil et al. found a pretherapeutic TLR of 2.2 or SUV_max_ of 16.4 to discriminate response in 60 liver metastases from NET undergoing PRRT, with non-responding lesions defined as either stable or progressive based on structural response criteria [26]. While rapid measurability and supposed stability over time predestine liver uptake as the inclusion threshold for RLT, quantitative validation of liver-derived threshold values has so far not been undertaken. This leaves an open question as to the extent lesions under the commonly applied thresholds respond to therapy. In our analysis we found a TLR of 1.76 to separate non-responding lesions from stable and responding lesions (AUC 0.83). Limitations to this approach must be pointed out, including possible bias in lesion selection and limited prognostic utility of calculated threshold values. As we observed, lesions with low TLR values (≤1.0) were still responsive to treatment in a significant fraction (45.2%) and remained non-progressive in the majority of cases (65.1%). This may support the notion that non-dominant tumor sites with low PSMA expression should not preclude patients from undergoing RLT. Yet, low overall PSMA expression in the bulk of patients’ disease burden is a risk factor for poor RLT outcomes, as previously shown. Seifert et al. evaluated PET/CT-derived total tumor volume in 85 RLT patients by assessing the lowest, average, and highest SUV_max_ values per patient at baseline. Patient groups with low average SUV_max_ values < 14.3 in the whole tumor burden had shorter overall survival following RLT, with 5.3 vs. 15.1 months (*p* < 0.001) [27].

Recently met consensus on PSMA PET/CT imaging suggests the adoption of a 4-point visual scoring scale (V-score) in addition to routine semiquantitative uptake values [11]. This approach allows for a rapid and scanner-independent stratification of disease avidity using blood-pool and normal organ uptake as reference landmarks. Similar scoring systems have previously been put in place for molecular imaging in NET (Krenning score) [28] and FDG-PET/CT response assessment in lymphomas (Deauville criteria) [29]. The role of a V-score in mCRPC patients undergoing RLT remains to be elucidated. Interestingly, in this study, lesions reverting to visual levels below the blood pool after RLT (V-score = 0) showed lower baseline uptake levels than lesions with higher posttreatment uptake (V-score 1–3). This result appears contradictory at first sight, while in line with findings from van der Sar et al., where lesions with imaging-based complete remission (iCR) after two RLT cycles had lower baseline SUV_max_ and SUV_peak_ values compared to other response categories [21]. As assessed by posttherapeutic visual scoring (Figure 6A), a considerable fraction of 230/505 (41.8%) lesions showing response to RLT still had high levels of PSMA expression (V-score ≥2) after treatment, thus maintaining targetability for further cycles of RLT in otherwise responding patients. A possible disadvantage of organ-derived thresholds is the potential variability of normal organ uptake under RLT, either by irradiation or by a change in tumor burden. The observed shift in normal uptake in the above-mentioned instances of discordant visual uptake increase and quantitative response is an anecdotal finding that may be attributed to a tumor-sink phenomenon which has been previously postulated and which calls for further investigation in dedicated cohorts [3].

In our study a large fraction, 550/718 (77%), of lesions included at baseline showed response to RLT. Concomitant disease progression was observed through emergence of new tumor sites in 11 otherwise responsive patients, as defined by the Prostate Cancer Working Group 3 criteria (PCWG 3) [30]. It is well acknowledged that single-lesion response is infrequently correlated with therapeutic outcome, rendering target lesion-based response assessment a limited tool for overall response assessment in mCRPC. It may be hypothesized that disease progression under RLT is more likely propagated by the rise of new tumor sites rather than non-responsiveness of existing lesions with intact PSMA expression. These new sites could be the result of either previously occult lesions or clonal expansion of resistant cancer cell phenotypes. In patients with high disease burden, volumetric tumor segmentation algorithms are under investigation and may play a role in future clinical routine [27,31].

Limitations of this study include its retrospective nature and limited population size, which inevitably impact on the strength of conclusions drawn. Potential selection bias of lesions at baseline cannot be ruled out, even though the adopted protocol incorporated both high-uptake lesions and lesions with minimal baseline uptake to represent a broad range of intra- and interindividual variability. Secondly, SUV_max_ decline was considered the decisive parameter for lesion response in accordance with modified response criteria for molecular response [32]. It must be acknowledged that PSMA uptake-derived response criteria are still subject to debate and so far not uniformly defined, which may impact the comparability of the presented results [10,33,34]. In small lesions that may have been subject to partial volume effects, single-lesion response could potentially be overestimated. Conversely, baseline uptake in small tumor lesions might have been underscored. Furthermore, it must be noted that a loss of PSMA expression can be a result of dedifferentiation and gain of FDG avidity, as previously described [35]. FDG PET/CT was not routinely available in pre- and posttherapeutic workup, thus this phenomenon could not be apprehended and remains to be addressed by future investigation.

## 5. Conclusions

Standard uptake parameters quantified by routine baseline [^68^Ga]Ga-PSMA-11 PET/CT are moderately associated with lesion response to [^177^Lu] Lu-PSMA-617. Lesions with uptake values below the liver uptake remain non-progressive in the majority of cases examined in this cohort. Non-dominant tumor sites with low PSMA expression should thus not preclude patients from undergoing RLT.

## Figures and Tables

**Table 1:** Baseline characteristics of treated patients.

Value	Normal Range	All Patients (n = 61)
Age		72 (67–78)
PSA (µg/L)	(<4)	69 (7–181)
Hemoglobin (g/L)	(13.5–17.5)	11.9 (10.9–13.3)
White blood cells (10^9^/L)	(3.92–9.81)	6.07 (4.84–7.64)
Platelets (10^9^/L)	(146–328)	231 (189–267)
eGFR (mL/min/1.73 m^2^)	(42–98)	85 (76–96)
Alkaline phosphatase (U/L)	(40–130)	82 (60–141)
LDH (U/L)	(<248)	242 (211–277)
Gleason score *		
<8		15 (27)
≥8		40 (73)
**ECOG performance status**		
1		29 (48)
2–3		9 (15)
**Tumor load**		
Low		10 (16)
Intermediate		14 (23)
High		37 (61)
**Sites of metastases**		
Bone		52 (85)
Lymph node		48 (79)
Visceral		12 (20)
Bone + Lymph node		39 (64)
Bone + Lymph node + Visceral		10 (16)
**Prior systemic therapies for mCRPC**		
Abiraterone		37 (61)
Enzalutamide		33 (54)
^223^Radium-dichloride		17 (28)
Docetaxel		28 (46)
Cabazitaxel		12 (20)

Data presented with median and interquartile range (IQR) or n (%) *: for available patients (*n* = 55), ECOG: Eastern Cooperative Oncology Group, PSA: prostate-specific antigen, eGFR: estimated glomerular filtration rate.

**Table 2:** Baseline characteristics of lesions (A), stratified by response (B). Data presented with median and interquartile range (IQR) or n (%). RL: responding lesions, SL: stable lesions, PL: progressive lesions, SUV: standardized uptake value, TLR: tumor-to-liver ratio.

**(A)**																	
**Site**		***n***				**SUV_max_**		**SUV_mean_**			**TLR**				**V-Score**		
															**1**	**2**	**3**
Bone		507				13.99 (8.15–22.11)		8.67 (5.06–13.59)			3.36 (2.05–5.65)				34	87	386
Lymph node		181				14.43 (8.64–25.95)		8.81 (5.51–16.93)			3.54 (1.88–7.27)				6	38	137
Visceral		22				13.00 (6.92–16.50)		8.15 (4.85–13.30)			2.59 (1.36–3.67)				1	4	17
Primary/prostate		8				17.52 (7.99–26.82)		10.22 (4.59–15.42)			3.76 (2.04–6.34)				1	1	6
All lesions		718				14.11 (8.25–23.01)		8.72 (5.09–14.39)			3.36 (1.98–5.70)				42	130	546
**(B)**																	
**Site**	**n (%)**					**SUV_max_**				**SUV_mean_**					**TLR**		
	**Total**	**RL**	**SL**	**PL**		**RL**	**SL**	**PL**		**RL**	**SL**	**PL**			**RL**	**SL**	**PL**
Bone	507	389 (77)	84 (17)	34 (7)		16.02 (10.02–23.8)	10.18 (6.29–15.74)	5.11 (4.21–8.61)		9.74 (6.03–14.96)	6.45 (3.92–9.8)	3.07 (2.46–5.12)			3.86 (2.32–6.30)	2.51 (1.68–3.70)	1.43 (0.76–2.36)
Lymph node	181	143 (79)	29 (16)	9 (5)		15.42 (9.55–29.73)	11.08 (8.97–21.58)	5.09 (4.72–6.36)		10.23 (5.97–18.93)	8.03 (5.89–13.67)	3.32 (2.57–4.13)			4.08 (2.2–8.86)	2.73 (1.48–3.95)	1.14 (0.97–1.54)
Visceral	22	12 (55)	7 (32)	3 (14)		15.13 (10.09–17.24)	11.58 (6.92–24.25)	5.44 (5.22–7.94)		10.75 (6.38–12.80)	6.35 (5.42–16.88)	4.29 (3.14–4.78)			2.88 (1.91–3.60)	3.43 (1.32–5.71)	1.31 (0.9–1.36)
Primary/ prostate	8	6 (75)	1 (13)	1 (13)		22.12 (15.79–28.65)	3.32	3.62		13.25 (8.88–15.90)	2.04	2.15			4.88 (3.31–7.11)	0.71	1.44
All lesions	718	550 (77)	121 (17)	47 (7)		16.01 (9.88–25.15)	10.82 (6.49–16.46)	5.11 (4.21–7.94)		9.88 (6.03–15.51)	6.62 (4.05–11.60)	3.18 (2.46–4.78)			3.89 (2.30–6.52)	2.55 (1.58–3.70)	1.36 (0.84–1.76)

**Table 3:** Lesion response stratified by various liver-derived threshold values. Data presented as n (%). RL: responding lesions, SL: stable lesions, PL: progressive lesions, TLR: tumor-to-liver ratio, PPV: positive predictive value, NPV: negative predictive value, OR: odds ratio, CI: confidence interval.

Threshold (Related to SUV_mean_ Liver)		Total	n (% of Total)			Predictive Value for PL		OR (95% CI) for Lesion Progression
			RL	SL	PL	PPV	NPV	
1.00	<1.00	42	19 (45)	9 (21)	14 (33)	33%	95%	9.74 (4.69–20.23), *p* < 0.001
	≥1.00	676	531 (79)	112 (17)	33 (5)			
1.50	<1.50	123	64 (52)	30 (24)	29 (24)	24%	97%	9.89 (5.28–18.52), *p* < 0.001
	≥1.50	595	486 (82)	91 (15)	18 (3)			
1.76	<1.76	155	85 (55)	35 (23)	35 (23)	23%	98%	13.39 (6.75–26.56), *p* < 0.001
	≥1.76	563	465 (83)	86 (15)	12 (2)			
2.00	<2.00	183	105 (57)	42 (23)	36 (20)	20%	98%	11.67 (5.80–23.48), *p* < 0.001
	≥2.00	535	445 (83)	79 (15)	11 (2)			

## Data Availability

The datasets analyzed and/or analyzed during the current study are available from the corresponding author on reasonable request.
